# Reduction of Polarization Field Strength in Fully Strained c-Plane InGaN/(In)GaN Multiple Quantum Wells Grown by MOCVD

**DOI:** 10.1186/s11671-016-1732-y

**Published:** 2016-11-25

**Authors:** Feng Zhang, Masao Ikeda, Shu-Ming Zhang, Jian-Ping Liu, Ai-Qin Tian, Peng-Yan Wen, Yang Cheng, Hui Yang

**Affiliations:** 1 Suzhou Institute of Nano-Tech and Nano-Bionics, Chinese Academy of Sciences (CAS), Suzhou, 215123 People’s Republic of China; 2 Key Lab of Nanodevices and Applications, Suzhou Institute of Nano-Tech and Nano-Bionics, Chinese Academy of Sciences (CAS), Suzhou, 215123 People’s Republic of China

**Keywords:** Polarization field, InGaN/(In)GaN multiple quantum wells, Photoluminescence, Strain relaxation, Reciprocal space mapping

## Abstract

The polarization fields in c-plane InGaN/(In)GaN multiple quantum well (MQW) structures grown on sapphire substrate by metal-organic chemical vapor deposition are investigated in this paper. The indium composition in the quantum wells varies from 14.8 to 26.5% for different samples. The photoluminescence wavelengths are calculated theoretically by fully considering the related effects and compared with the measured wavelengths. It is found that when the indium content is lower than 17.3%, the measured wavelengths agree well with the theoretical values. However, when the indium content is higher than 17.3%, the measured ones are much shorter than the calculation results. This discrepancy is attributed to the reduced polarization field in the MQWs. For the MQWs with lower indium content, 100% theoretical polarization can be maintained, while, when the indium content is higher, the polarization field decreases significantly. The polarization field can be weakened down to 23% of the theoretical value when the indium content is 26.5%. Strain relaxation is excluded as the origin of the polarization reduction because there is no sign of lattice relaxation in the structures, judging by the X-ray diffraction reciprocal space mapping. The possible causes of the polarization reduction are discussed.

## Background

InGaN/(In)GaN multiple quantum well (MQW) has been successfully used in light emitting devices with wavelength ranging from violet to green. However, for typical InGaN-based devices which are grown on c-plane, it is well known that there exists a large electric field caused by spontaneous and piezoelectric polarization effect, which affects the electrical and optical properties of the devices significantly [1, 2]. The polarization effect leads to inclined quantum wells, from which the emission wavelength is red-shifted. Due to the large polarization field strength in the quantum wells (usually in the order of MV/cm), the quantum levels in the triangular wells are quite different from the case of the rectangular wells. It is worth noting here that the change in the polarization field can be reflected in the emission wavelength. The theoretical polarization field in the MQWs was reported by Bernardini et al. [3, 4], which is widely accepted.

The polarization field can be screened by applied voltage, free carriers or charged defects, and strain relaxation. By applying reverse bias on the structures, the polarization field can be canceled, and the field strength can be then deduced from the change of applied voltage or emission wavelength. Based on this, several experimental methods were put forward to determine the polarization field in quantum well structures including photoluminescence (PL) [5], electroreflectance [6], electrotransmission [7], photocurrent [8], and Raman scattering [9]. It has been reported that the polarization field can be screened by doping, defects, and carrier injection [10–13], therefore resulting in a blue-shifted emission wavelength. The strain relaxation might lead to a reduction in polarization field, because the piezoelectric polarization is determined by the strain status of the epi-layers. Once the strain is relaxed, the piezoelectric polarization would be weakened accordingly.

In this paper, the polarization strengths in quantum well structures with indium content varying from 14.8 to 26.5% are experimentally investigated through photoluminescence and high-resolution X-ray diffraction (HR-XRD). The emission wavelengths of MQWs are theoretically determined by considering various effects comprehensively. The PL wavelength is found to be much shorter than expected when the indium composition is higher than 17.3%, which is attributed to the reduction of polarization field. The polarization fields in MQWs with highest indium composition are found to be only ~23% of the theoretically values. In order to investigate the origin of the weakening of polarization, reciprocal space mapping (RSM) was conducted. The result turns out that there is no sign of lattice relaxation in the MQWs studied in this paper.

## Methods

### Sample Description

**Fig. 1:**
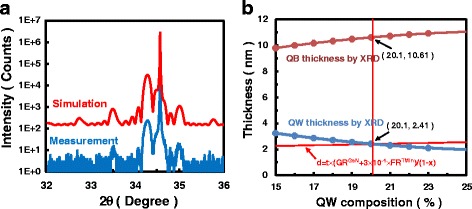
**a** The omega/2theta scan of MQW sample. **b** The dependence of QW and QB thickness on composition determined by XRD and growth rate

Five periods of InGaN/(In)GaN MQWs with various indium composition in the QWs were grown on sapphire substrate by Aixtron 6 × 2 in. close coupled showerhead metal-organic chemical vapor deposition reactor. A 30-nm GaN nucleation layer was deposited on the sapphire substrate, and then two kinds of under-layers were grown. One is a 4-μm Si-doped GaN layer grown at 1007 °C, the other is a 1-μm Al_0.06_Ga_0.94_N inserted in the 4-μm GaN grown at the same temperature. Unintentionally doped InGaN/(In)GaN quantum wells were grown continuously at 735 °C onto the under-layer. Triethylgallium (TEGa), trimethylindium (TMIn), and ammonia (NH_3_) were used as the precursors of gallium, indium, and nitrogen, respectively. The indium contents in the quantum wells and quantum barriers (QBs) were controlled by changing the TMIn flow rate during the growth. The samples with InGaN QBs were capped by 2 nm GaN grown at the same temperature as MQWs to protect the material surface. The TEGa molar flow rate was fixed at 4.95 μmol/min, and the TMIn molar flow rate was set as 6.51, 10.75, 9.90, 13.44, and 17.68 μmol/min for sample A to E, respectively. Table 1 shows the detailed structures of some selected samples. The averaged composition is calculated assuming periodic structure. HR-XRD was used to determine the composition and thickness of both QWs and QBs. Generally speaking, it is difficult to determine quite accurately the thickness and composition of the QWs and QBs, because XRD can only give the averaged composition of the MQWs and the thickness of one period. The indium composition can vary due to growth temperature fluctuation of the substrate surface during MOCVD growth. Moreover, it has been reported that in close coupled showerhead reactors, the growth rate of GaN in InGaN layers can be enhanced by increasing TMIn flow [14], which makes the determination of InGaN composition and thickness much complex. This enhanced growth rate determined experimentally was taken into consideration in the HR-XRD fitting to accurately determine the thickness and composition of each sample. Figure 1a shows the result of HR-XRD omega-2theta scan as an example. Figure 1b shows the determination of thickness and composition of QWs and QBs. A series of QW and QB thicknesses are derived from XRD fitting for the given indium content in the QW assuming fully strained conditions. Good fitting can be obtained with each set of parameters. The thickness of QW determined by the growth rate and growth time is also shown. The thickness of InGaN QW can be described by *d* = *t*(*GR* ^GaN^ + 3.0 × 10^− 5^ × *FR* ^TMIn^)/(1 − *x*), in which *t* denotes the growth time, *GR* ^GaN^ is the growth rate of GaN when the TMIn flow rate is zero, *FR* ^TMIn^ is the flow rate of TMIn, and *x* is the composition in InGaN. By combining the XRD and growth behavior altogether, the QW and QB thickness and composition can be accurately determined.

**Table 1:** Sample structures determined by growth parameters and HR-XRD

Sample	*x*_QW_ (%)	*d*_QW_ (nm)	*x*_QB_ (%)	*d*_QB_ (nm)	$\overline{\mathrm{x}}$ (%)
A	14.8	2.90	0.0	12.36	2.81
B	17.3	2.48	0.0	9.90	3.47
C	19.5	2.10	3.8	10.65	6.37
D	23.2	2.30	1.2	10.06	5.29
E	26.5	2.62	0.0	9.94	7.32

A 405-nm cw laser was used in the room temperature (RT) PL measurement. The excitation power density was ~1 W/cm^2^. The emitted light was dispersed by a 1-m focal length single grating monochromator and detected by a CCD camera in the range of QW transitions. The emission wavelength was determined by fitting the spectra with a single peak Gaussian function. RMS was measured around the asymmetric $\left(10\overline{1}5\right)$ Bragg reflection for sample A, C, D, and E to examine the strain relaxation in the MQWs.

### Theoretical Calculation

In the (001) zinc-blende material system without polarization field, once the square quantum well structure is determined, the quantum levels can be accurately calculated. However, in the III-nitrides wurtzite materials, especially in indium containing material, it is more difficult to determine the quantum levels accurately due to the strong polarization field. In this paper, we proposed a comprehensive model to estimate the emission wavelength of InGaN MQWs. The emission wavelength is determined not only by the indium composition, but also by many other effects including strain, Stokes-like shift, quantum-confined Stark effect, and quantum levels as shown in Eq. 1.

1 $$ \raisebox{1ex}{$hc$}\!\left/ \!\raisebox{-1ex}{$\lambda $}\right.={E}_{\mathrm{g}}+{E}_{\mathrm{strain}}-{E}_{\mathrm{QCSE}}-{E}_{\mathrm{Stokes}}+{E}_{\mathrm{e}}+{E}_{\mathrm{h}}. $$

*E*_g_ is the bandgap of InGaN calculated by interpolation of binary GaN and InN, which is 3.435 and 0.71 eV respectively, assuming a bowing factor of 1.4. *E*_strain_ is the energy shift caused by strain. *E*_QCSE_ is the energy shift caused by the polarization field in the quantum well as denoted by *qd*_w_ *F*_w_, where *q* is the electron charge, *d*_w_ is the quantum well thickness, and *F*_w_ is the polarization field in the quantum well. *E*_Stokes_ is the Stockes-like shift energy which is assumed to be proportional to the indium composition. *E*_e_ and *E*_h_ are the quantum levels of electron and hole relative to the conductive band minimum and valence band maximum in the quantum well. Both *E*_QCSE_ and quantum levels are related to the polarization field in the wells. Here, we neglected the effects of bandgap normalization and band-filling, since the photo-excited carrier density in this study is relatively low around 10^17^ cm^−3^. More detailed consideration of each effect can be found in Ref 15. The theoretical polarization strength is calculated according to the non-linear polarization reported by Fiorentini and Barnardini et al. [3]. In MQW structures, the polarization field is calculated assuming periodic boundary condition:

2 $$ {F}_j=\frac{{\displaystyle {\sum}_k}{l}_k{\boldsymbol{P}}_k/{\varepsilon}_k-{\boldsymbol{P}}_j{\displaystyle {\sum}_k}{l}_k/{\varepsilon}_k}{\varepsilon_j{\displaystyle {\sum}_k}{l}_k/{\varepsilon}_k}, $$

in which *l*_*k*_, *P*_*k*_, *ε*_*k*_ represents for the thickness, polarization, and dielectric constant of the *k*th layer. The calculated polarization field strengths in the QWs and QBs are shown in Table 2. The quantum levels of electron and hole in the QWs are also calculated by self-consistent Poisson-Schrodinger calculation, assuming 100% polarization strength in the QWs. It is noted here again that the quantum levels are strongly dependent on the polarization field in the QWs. When the polarization field changes, the quantum levels have to change accordingly.

**Table 2:** Theoretical polarization field strengths, quantum levels, and theoretical wavelengths assuming 100% polarization field

Sample	F_w_^100%^ (MV/cm)	F_B_^100%^ (MV/cm)	λ_theoretical_ (nm)	λ_measured_ (nm)
A	−2.01	0.471	450.4	451.0
B	−2.35	0.588	455.4	456.0
C	−2.30	0.454	453.8	438.1
D	−3.13	0.716	510.0	461.7
E	−3.42	0.901	570.9	469.5

The Stokes-like shift energy in the 16% InGaN quantum wells was determined to be 0.08 eV experimentally [15]. When the indium content increases, the Stokes-like shift energy also increases due to larger indium composition fluctuation. It is assumed that the Stokes-like shift energy is proportional to the indium composition in this paper, which is consistent with the reported results [16, 17]. Combining all the effects listed above, the theoretical emission wavelengths can be determined for each sample.

## Results and Discussion

**Fig. 2:**
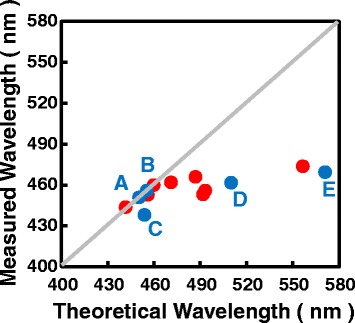
The comparison between the measured and theoretical wavelengths

**Fig. 3:**
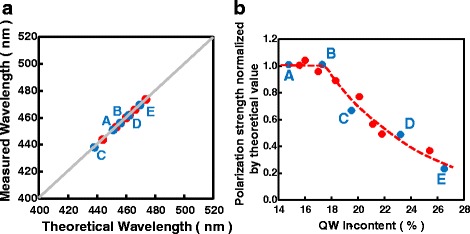
**a** The comparison between measured and theoretical wavelengths considering reduced polarization field. **b** The polarization ralative to the theoretical values

Figure 2 shows the comparison between the measured and the theoretical wavelengths. Here, the theoretical wavelengths are obtained assuming 100% theoretical polarization strength. It is clear that for sample A and B with indium composition lower than 17.3%, the measured wavelengths agree well with the theoretical results. However, for sample C–E with indium composition higher than 17.3%, the measured values are much smaller than the theoretical ones. The discrepancy can be as large as 470 meV for sample E. According to Eq. 1, this discrepancy can be ascribed to a decreased Stokes-shift energy, an increased quantum energy, or a reduced polarization field. Among these candidates, lower Stokes-shift energy cannot be possible because we used a moderate value obtained experimentally. The quantum energy is dependent weakly on the quantum well thickness and material parameters such as effective masses $\left(\propto {m}^{*-\frac{1}{3}}\right)$, but strongly on the polarization field. Therefore, the change in polarization field is the only, or at least, main reason responsible for this large discrepancy. The emission wavelength is calculated again taking into consideration the weakened polarization field as shown in Fig. 3. For samples A, B, and C, in which the indium compositions are smaller than 17.3%, the polarization field strength remain to be 100% of theoretical values. However, for other samples, which have higher indium composition, the polarization field decreases significantly; as shown in Fig. 3b, the minimum polarization ratio is around 23% for the highest indium content of 26.5%.

**Fig. 4:**
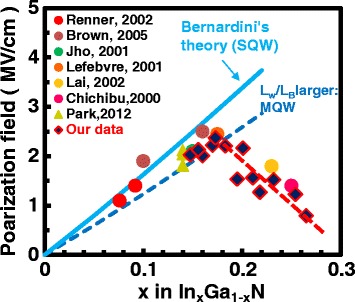
The reported and our data of polarization field

The reported polarization field strengths in various SQWs and MQWs in the literature are shown together with our data for the MQWs in Fig. 4, in which the theoretical values are shown also. The blue solid line shows the theoretical value after Bernardini et al. for single quantum wells (SQW). While the blue broken line is the theory for MQWs, which is smaller than the case in SQW due to the larger thickness ratio of QW and QB. Both the reported [18–23] and our data are in good agreement with the theory when the composition is relatively lower. However, when the composition is larger (>17.3% ), the polarization field is reduced relative to the theoretical value.

**Fig. 5:**
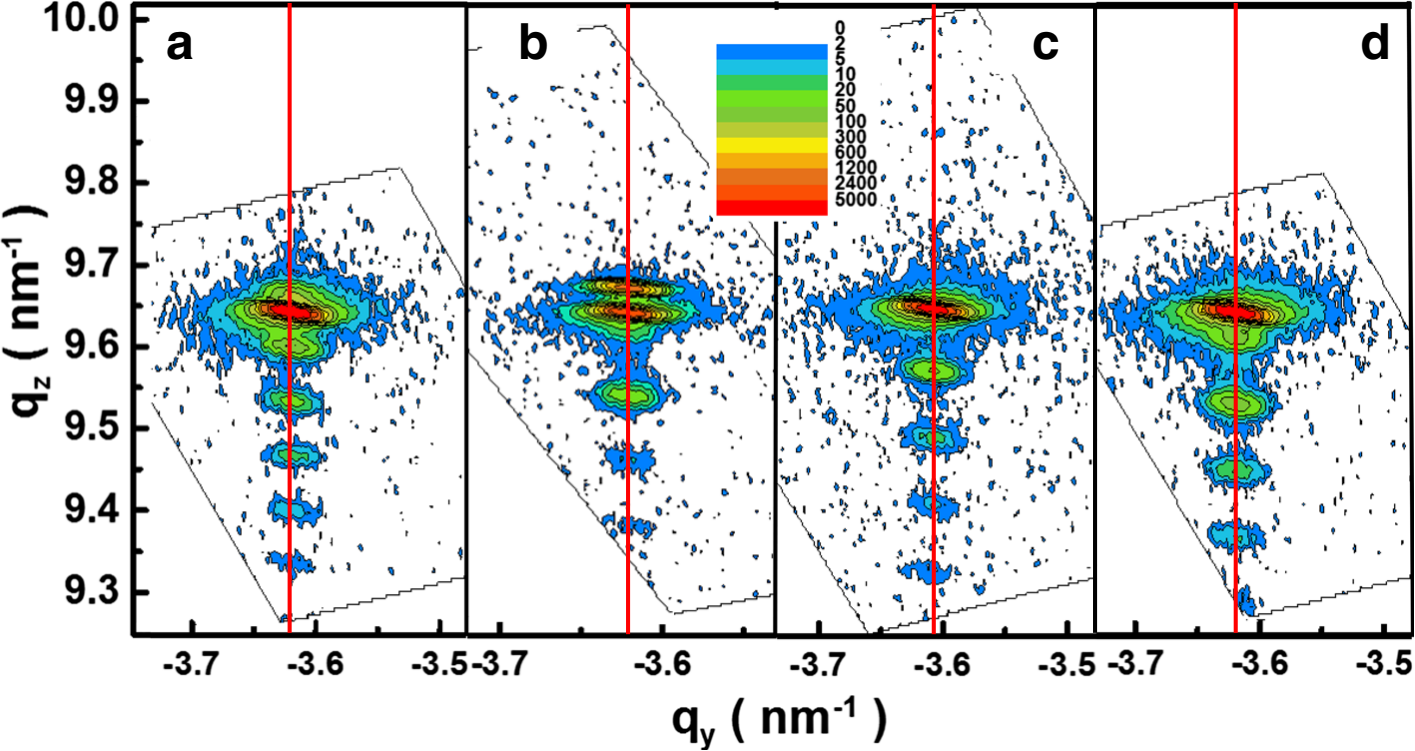
RSM around the asymmetric (1015) Bragg reflection for sample A (**a**), C (**b**), D (**c**), and E (**d**)

**Fig. 6:**
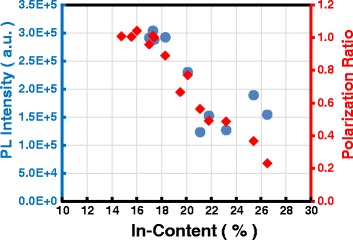
PL intensity and polarization ratio for some samples

Generally speaking, the relaxation of strain is thought to be the origin of reduction of piezoelectric polarization. In order to clarify the origin of the reduction of polarization field, the lattice relaxation is checked through asymmetric RSM for selected samples. The reported critical thickness for In_0.16_Ga_0.86_N is only ~10 nm [24]. Although the total thickness of our QWs is larger than the reported critical layer thickness of InGaN, the averaged composition is far below. As is shown in Fig. 5, there is no sign of lattice relaxation in all the samples measured. This excludes the macroscopic lattice relaxation as the origin of the polarization reduction. Because the polarization of III-nitrides originates from the displacement of the charge centers in wurtzite lattice, any disorders in the lattice can affect the polarization strength. Here we speculate that several causes are responsible for the polarization reduction. One is the point defects or impurities in the epi-layers and interfaces. If some defects such as vacancies or interstitials or impurities exist, the displacement of atoms can be distributed over several neighboring atoms, leading to a significant change in the polarization. Moreover, the charged defects or impurities can also screen the field due to coulombic interaction. It is difficult to estimate the density of the defects or impurities at the present stage, because the type of defects or impurities remains unknown. Indium fluctuation can be another candidate of the causes. It has been observed that the polarization screening in indium-rich emission centers can result in a blue-shift in transition energies by up to 400 meV [25], which indicates quite different microscopic strain status in some of the indium-rich centers. It has been reported that indium segregation can happen near the threading dislocation cores [26], giving rise to the disorder of the lattice. Because our MQWs were grown on sapphire substrate, the threading dislocation density was estimated to be the about 2 × 10^8^ cm^−3^ for screw and mixed-types and 1–2 × 10^9^ cm^−3^ for edge type. Indium fluctuation could be further enhanced by such a high threading dislocation density. The integrated PL intensity for some samples is shown in Fig. 6 together with the polarization ratio. A similar tendency can be found in the PL intensity and polarization ratio, suggesting possible causes of the reduction in polarization. Higher composition results in higher defect density, corresponding to lower PL intensity.

## Conclusions

The polarization fields in the MQWs with indium composition ranging from 14.8 to 26.5% are investigated comprehensively. It is found that when the indium content is smaller than 17.3%, 100% theoretical polarization field strength can be maintained, while when the indium content is larger than 17.3%, the polarization field strength decreased significantly. This reduction is proved not due to the macroscopic lattice relaxation in the epitaxy layers, but probably the increased point defects or impurities as well as larger indium fluctuation in the InGaN quantum well layers.

## Abbreviations

HR-XRD: High-resolution X-ray diffraction
MQW: Multiple quantum well
NH_3_: Ammonia
PL: Photoluminescence
QB: Quantum barrier
QCSE: Quantum confined Stark effect
RSM: Reciprocal space mapping
TEGa: Triethylgallium
TMIn: Trimethylindium
